# Enhanced convective heat transfer using graphene dispersed nanofluids

**DOI:** 10.1186/1556-276X-6-289

**Published:** 2011-04-04

**Authors:** Tessy Theres Baby, Sundara Ramaprabhu

**Affiliations:** 1 Alternative Energy and Nanotechnology Laboratory (AENL), Nano Functional Materials Technology Centre (NFMTC), Department of Physics, Indian Institute of Technology Madras, Chennai 600036, India

## Abstract

Nanofluids are having wide area of application in electronic and cooling industry. In the present work, hydrogen exfoliated graphene (HEG) dispersed deionized (DI) water, and ethylene glycol (EG) based nanofluids were developed. Further, thermal conductivity and heat transfer properties of these nanofluids were systematically investigated. HEG was synthesized by exfoliating graphite oxide in H_2 _atmosphere at 200°C. The nanofluids were prepared by dispersing functionalized HEG (f-HEG) in DI water and EG without the use of any surfactant. HEG and f-HEG were characterized by powder X-ray diffractometry, electron microscopy, Raman and FTIR spectroscopy. Thermal and electrical conductivities of f-HEG dispersed DI water and EG based nanofluids were measured for different volume fractions and at different temperatures. A 0.05% volume fraction of f-HEG dispersed DI water based nanofluid shows an enhancement in thermal conductivity of about 16% at 25°C and 75% at 50°C. The enhancement in Nusselts number for these nanofluids is more than that of thermal conductivity.

## Introduction

Most industries use conventional fluids like deionized (DI) water, ethylene glycol (EG), transformer oil, etc., as heat transfer fluids. The efficiency of the heat transfer fluid determines the productivity and lifetime of the equipments, electronic circuits, machines, etc. The efficiency of the heat transfer fluids can be increased by enhancing the thermal conductivity and heat transfer properties. Conventional fluids have low thermal conductivity compared to solid counter parts. Therefore, solid particles with high thermal conductivity are generally added to these fluids to enhance their thermal conductivity. However, the addition of macro- and micro-sized particles can create problems like agglomeration and sedimentation. To avoid these problems Choi, Eastman, and co-workers [1,2] introduced a new type of fluid called nanofluid wherein nanomaterials are dispersed in base fluids like water or EG. Subsequently many research groups have worked on the thermal conductivity and heat transfer mechanism of different nanomaterials dispersed nanofluids. Several groups have shown enhancement in thermal conductivity with Al_2_O_3 _and CuO nanoparticles dispersed water and EG based nanofluids [3-5]. The enhancement in thermal conductivity depends on several parameters like, size and shape of the nanomaterials, pH of the base fluid, temperature of the fluid, presence of additives, volume fraction of the nanomaterials, etc.

Similar to thermal conductivity, heat transfer mechanism also plays a crucial role in nanofluids. The use of nanofluids having good heat transfer properties reduces the size of the entire unit thereby increases the efficiency of the unit. Hence, it is necessary to determine the heat transfer performance of various nanofluids under dynamic flow conditions apart from steady state thermal conductivity measurements. The heat transfer measurements have been carried out for different flow conditions, laminar flow, and turbulent flow by several groups. Yang et al. [6] studied the heat transfer performance of several nanofluids under laminar conditions in a horizontal tube heat exchanger. Heris et al. [7] found heat transfer enhancement as high as 40% with Al_2_O_3 _particles. However, there is not much work on the heat transfer mechanism of carbon based nanofluids except a few on carbon nanotubes (CNTs) [8].

Recently, the two-dimensional one carbon atom thick graphene was found to exhibit high crystal quality and ballistic electron transport at room temperature. Theoretical study of thermal conductivity on graphene suggests that it is having unusual thermal conductivity [9,10]. Following this, Balandin et al. [11] measured experimentally the thermal conductivity of about 5300 W/mK for a single layer graphene from the dependence of the Raman G peak frequency on the excitation laser power. The thermal conductivity of single layer graphene is higher than that of CNTs.

To our knowledge, there is no work on the heat transfer properties of graphene based nanofluids. In the present work, we have synthesized graphene dispersed nanofluids and studied its thermal conductivity and heat transfer properties. The nanofluids were prepared by taking DI water and EG as base fluids.

## Experimental methods

### Materials

Graphite (99.99%, 45 μm) was purchased from Bay Carbon, Inc, USA. All other reagents like sulfuric acid, nitric acid, sodium nitrate, potassium permanganate, hydrogen peroxide, and ethylene glycol were analytical grade. DI water was used throughout the experiment. Graphite oxide (GO) was prepared from graphite using Hummers method [12]. Briefly, 2 g of graphite was treated with 46 ml of sulphuric acid in an ice bath. One gram of sodium nitrate was added to the above solution slowly, followed by the addition of 6 g of potassium permanganate. At room temperature, specific quantity of water was added to the above mixture. After 15 min the suspension was further treated with hydrogen peroxide and was filtered. Finally the filter cake was washed with copious quantity of DI water. At last, the suspension was filtered and dried in vacuum oven at 40°C for 8 h. The dried GO was used for synthesizing hydrogen exfoliated graphene (HEG). Exfoliation of GO was done in hydrogen atmosphere at 200°C as reported previously [13]. Functionalization of HEG was done by treating as synthesized HEG with conc. H_2_SO_4_:HNO_3 _in the ratio 3:1. The acid-HEG mixture was ultrasonicated for about 3 h at room temperature. After 3 h the sample was washed several times with DI water, filtered and dried in vacuum.

### Characterization techniques

The samples were characterized with different characterization techniques. Powder X-ray diffraction (XRD) studies were carried out using a PANalytical X'PERT Pro X-ray diffractometer with Nickel-filtered Cu K_α _radiation as the X-ray source. The pattern was recorded in the 2θ range of 5° to 90° with a step size of 0.016°. The Raman spectra were obtained with a WITEC alpha 300 Confocal Raman spectrometer equipped with Nd:YAG laser (532 nm) as the excitation source. Identification and characterization of functional groups were carried out using PerkinElmer FT-IR spectrometer in the range 500-4000 cm^-^^1^. Digital photograph has been taken with a Canon Power Shot A590 IS 8 Megapixel camera with 4 × optical zooming. Field emission scanning electron microscopy (FESEM) and transmission electron microscopy (TEM) images were obtained using, FEI QUANTA and JEOL TEM-2010F instruments, respectively. Nanofluid was prepared by dispersing a known amount of f-HEG in the base fluid by ultrasonication (30-45 min). Thermal conductivity of the suspension was measured using KD2 pro thermal property analyzer (Decagon, Canada). The probe sensor used for these measurements were of 6 cm in length and 1.3 mm in diameter. In order to study the temperature effect on thermal conductivity of nanofluid a thermostat was used. Electrical conductivity of the nanofluid was measured using ELICO Ltd CM 183, EC-TDS meter.

**Figure 1:**
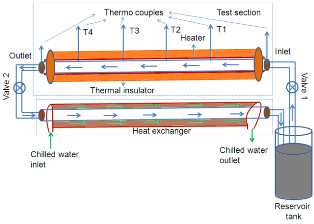
**Schematic of the heat transfer set up**.

The convective heat transfer mechanism was studied using an indigenously fabricated setup. The schematic of the setup is shown in Figure 1. It consists of a flow loop, a heat unit, a cooling part, and a measuring and control unit. The flow loop included a pump with flow controlling valve system, a reservoir and a test section. A straight stainless steel tube with 108 cm length and 23 mm inner diameter was used as the test section. The whole test section was heated by a copper coil linked to an adjustable DC power supply. There was a thick thermal isolating layer surrounding the heater to obtain a constant heat flux condition along the test section. Four T-type thermocouples were mounted on the test section at axial positions in mm of 298 (T1), 521 (T2), 748 (T3), and 858 (T4) from the inlet of the test section to measure the wall temperature distribution, and two further T-type thermocouples were inserted into the flow at the inlet and exit of the test section to measure the bulk temperatures of nanofluids. Cooling part is to cool down the nanofluid coming out from the outlet of test section.

## Results and discussion

### XRD and Raman analysis

**Figure 2:**
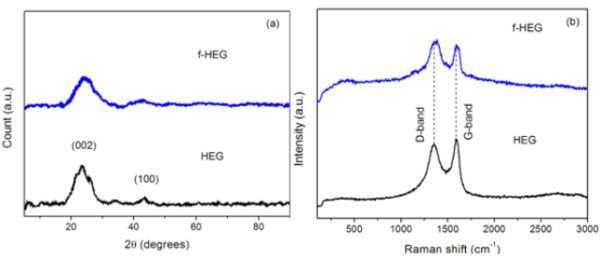
**Crystallographic study (a) X-ray diffractogram and (b) Raman spectra of HEG and f-HEG**.

The crystallinity of the samples was studied using XRD. Figure 2a shows the X-ray diffractogram of HEG and f-HEG. The characteristic (002) plane in graphite at approximately 26° is shifted to approximately 24° in HEG. This is same in all graphene prepared by different exfoliation techniques [14,15]. There is not much difference in XRD of f-HEG and HEG except the broadening of the (002) peak. After vigorous acid treatment, the layers might have separated further and that may be the reason for this broadening [16]. The functionalization and defects on the carbon based materials can be sought out by Raman spectroscopy [17]. Figure 2b shows the Raman spectrum of HEG and f-HEG. In HEG, the peak around 1588 cm^-1 ^corresponds to the G-band and the peak around 1356 cm^-1 ^corresponds to D-band. The D- and G-band represent the sp^3 ^and sp^2 ^hybridization of carbon atoms present in the sample, respectively. In the case of f-HEG, G-band as well as D-band shifted to higher wave number side and also broadened with respect to HEG peak positions. G-band has a broad peak centered around 1591 cm^-1 ^and D-band has a peak centered around 1371 cm^-1^. The ratio of the D-band intensity to G-band intensity in f-HEG is higher than that of HEG. The increase in the relative intensity of the disordered mode can be attributed to the increased number of structural defects and to the sp^3 ^hybridization of carbon for chemically induced disruption of the hexagonal carbon order after acid treatment. Acid treatment created some functional groups at the edges of the graphene sheets which helped for the proper dispersion of f-HEG on water and EG. The presence of functional groups may be the reason for broadening of the D-band peak.

### FTIR study and digital photograph

**Figure 3:**
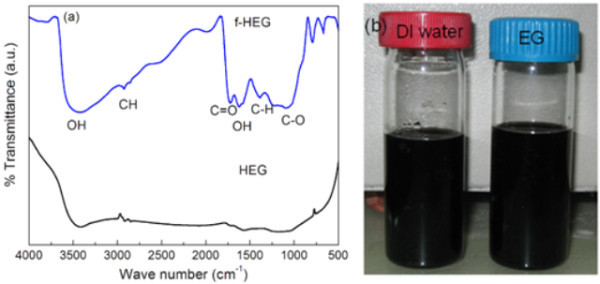
**Spectral analysis (a) FTIR spectrum of HEG and f-HEG; (b) Digital photograph nanofluid made up of f-HEG in DI water and EG**.

The effect of acid treatment and attachment of functional groups were further confirmed with FTIR. Figure 3a shows the FTIR spectra of HEG and f-HEG. During the heat treatment in H_2 _atmosphere, most of the oxygen containing functional groups has been removed from HEG. So the functional groups are not dominant in FTIR of HEG. After acid treatment, functional groups are formed at the plane and edges of the sheets. The peaks at around 3442 and 1625 cm^-^^1 ^are due to OH functional groups. A small doublet peak of CH_2 _(2922 and 2860 cm^-^^1^) and CH at 1365 cm^-^^1 ^are present both in HEG and f-HEG. The peaks at 1720 and 1380 cm^-^^1^can be assigned to the C = O and C-O stretching vibrations of COOH. These functional groups help graphene sheets to interact with water molecules and disperse properly. Figure 3b shows the digital photograph of f-HEG dispersed DI water and EG based nanofluid after 2 months of the nanofluid preparation. Even after 2 months of preparation no sedimentation was observed.

### Morphology of graphene sheet

**Figure 4:**
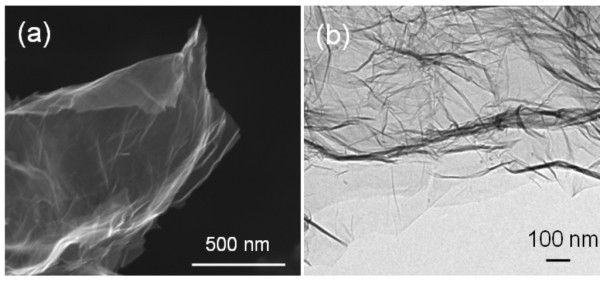
**Microscopy study (a) Field emission scanning electron microscopy and (b) transmission electron microscopy images of HEG**.

Figure 4a shows the FESEM image of as-synthesized HEG taken by putting a small amount of powder sample on carbon tape. The image shows a large area of transparent graphene sheet with rough and soft wrinkled surface morphology. Transmission electron microscopy is also a powerful technique used extensively to provide definitive identification of graphene materials. The sample preparation was done by depositing a drop of ethanol dissolved HEG on Cu grid. Figure 4b clearly shows the wrinkles on the surface and folding at the edges of HEG sheets.

### Thermal conductivity study of graphene nanofluid

**Figure 5:**
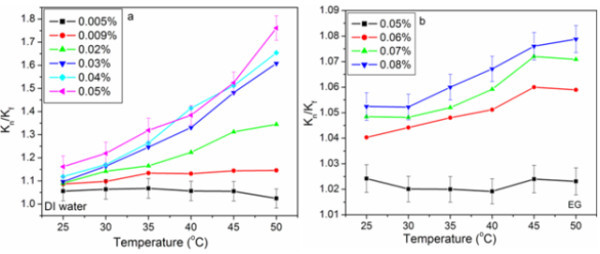
**Normalized thermal conductivity of f-HEG dispersed**. **(a) **DI water and **(b) **EG based nanofluids for different volume fractions and at varying temperatures.

The proper functionalization helped to make well-dispersed HEG nanofluid. Figure 5a shows the normalized thermal conductivity (*K*_n_/*K*_f_) of f-HEG dispersed DI water based nanofluid as a function of temperature for different f-HEG volume fractions. All the measurements were carried out for low volume fractions so as to keep the viscosity of the fluid at a minimum level. For DI water based f-HEG nanofluids, the range of volume fractions used was from 0.005 to 0.05%. The percentage enhancement in thermal conductivity was calculated using the relation ((*K*_n _- *K*_f_) × 100)/*K*_f_, where '*K*_f_' was the thermal conductivity of base fluid and '*K*_n_' was that of nanofluid. For 0.05% volume fraction, the enhancement in thermal conductivity is about 16% at 25°C and about 75% at 50°C. The enhancement is less than 10% for 0.005% volume fraction. It is clear from the graph that the thermal conductivity increases with increasing temperature and volume fractions. According to Das et al. [4], in nanofluid the main mechanism of thermal conductivity enhancement can be thought as the stochastic motion of the nanoparticles. This Brownian-like motion will be dependent on fluid temperature and so the huge enhancement in thermal conductivity with temperature is quite explicable. At low temperature this motion was less significant giving the characteristics of normal slurries which rapidly changed at elevated temperature bringing more nanoeffect in the conducting behavior of the fluid. The error bar is shown only for low and high volume fractions.

Figure 5b shows the normalized thermal conductivity of f-HEG dispersed EG based nanofluids with varying temperatures and volume fractions. The thermal conductivity of EG based nanofluids did not show much enhancement for low volume fractions. Till around 0.05% volume fraction there was no enhancement in thermal conductivity. Thermal conductivity started increasing from 0.05% volume fraction onwards. For 0.08% the enhancement was about 1% at 25°C and about 5% at 50°C. This low enhancement in thermal conductivity may be due to the high viscosity of EG. Even though the enhancement in thermal conductivity of EG based nanofluids with f-HEG is low, it is slightly higher than that of CNT dispersed EG [18].

**Figure 6:**
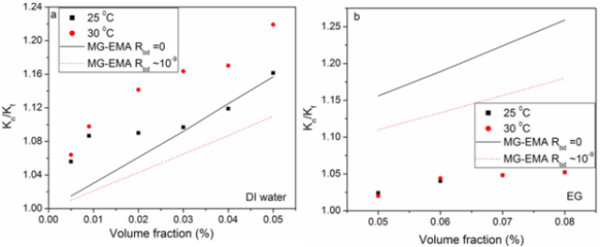
**Correlation of experiment with theory**. The enhancement of effective thermal conductivity of *K*_n_/*K*_f _as a function of volume fraction correlated for Maxwell-Garnet effective medium approximation (MG-EMA) for **(a) **DI water and **(b) **EG based f-HEG dispersed nanofluids.

The conventional theoretical models on thermal conductivity of nanoparticles suspended fluids does not consider particle size, shape, the distribution, and the motion of dispersed particles, while only thermal conductivities of base fluid and particles, and volume fraction of article. Maxwell was the man who first investigated the thermal conductivity of liquid suspensions analytically [19]. Later, Hamilton and Crossser modified Maxwell's model by taking in to consideration of geometry of particles [20]. In 1987 Hasselman and Johnson [21] modified Maxwell's model by including the interfacial thermal resistance (Kapitza resistance, *R*_bd_). The resulting theoretical prediction for the effective thermal conductivity (*K*) enhancement of the particle-in-liquid colloidal suspensions is given by,(1)

where α = 2 *R*_bd_*K*_f _*/d*, *d *is the average particle diameter, *R*_bd _is the interfacial thermal resistance, *K*_f _and *K*_p _are the thermal conductivity of base fluid and particles, respectively. This is also called Maxwell-Garnett type effective medium approximation (MG-EMA). In the absence of thermal boundary resistance (*R*_bd _= 0), the above equation reduces to Maxwell's model. The results are shown in Figure 6a,b for DI water and EG, respectively. The thermal conductivity is correlated with lower and upper bounds of *R*_bd_. The lower and upper bound MG-EMA correlation is almost matching for the DI water based nanofluid at 25°C. When the temperature increases the thermal conductivity is going away from the correlated values. But in the case of EG based nanofluids the calculated value is very much less than the correlated value. This suggests that there are other mechanisms contribute to the thermal conductivity of EG based f-HEG dispersed nanofluids.

### Electrical conductivity of f-HEG dispersed nanofluids

**Figure 7:**
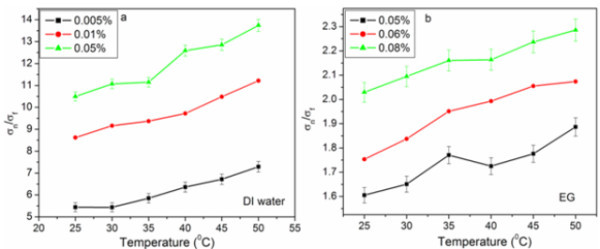
**Normalized electrical conductivity of f-HEG dispersed (a) DI water and (b) EG based nanofluids for different volume fractions and varying temperatures**.

Finally electrical conductivity was also measured for some volume fractions of f-HEG dispersed nanofluid. Figure 7a shows the normalized electrical conductivity (σ_n_/σ_f_) for three different volume fractions at varying temperature in DI water based nanofluid. 'σ_n_' represents the electrical conductivity of nanofluid and 'σ_f_' that of base fluid. The graph suggests that like thermal conductivity, electrical conductivity also increased with increase in volume fraction and increase in temperature. Similar trend was observed for f-HEG dispersed EG nanofluids also. Figure 7b shows the normalized electrical conductivity of f-HEG dispersed EG based nanofluid for three different volume fractions at varying temperature. The experiments were repeated several times and the error in measurements was less than 4%.

### Convective heat transfer

The heat transfer coefficient, (*h*) is a macroscopic parameter describing heat transfer when a fluid is flowing across a solid surface of different temperature. It is not a material property. The convective heat transfer coefficient is defined as(2)

where *x *represents axial distance from the entrance of the test section, *q *is the heat flux, *T*_s _is the measured wall temperature, and *T*_f _is the fluid temperature decided by the following energy balance:(3)

where *c*_*p *_is the heat capacity, *M *is the mass flow rate, and *E*(*x*) is the energy at position *x*. Equation 3 is based on an assumption of zero heat loss through the insulation layer.(4)

And mass flow rate can be calculated using the relation,(5)

where *u *is the velocity of flow, *A *is the area of cross-section, and ρ is the density of fluid. Reynolds number is defined as *Re *= ρuD/μ and the Prandtl number is defined as *Pr *= ν/α, where μ is the fluid dynamic viscosity, ν is the fluid kinematic viscosity, and α is the fluid thermal diffusivity.

#### Validity of the experimental setup with DI water

**Figure 8:**
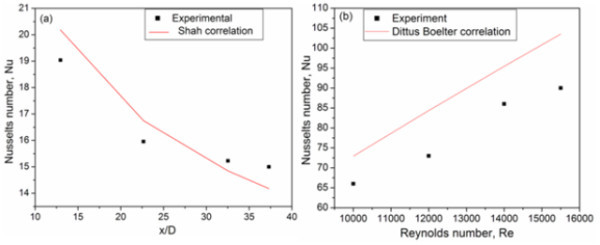
**Validity of the experimental heat transfer setup for (a) low (Shah correlation) and (b) high (Dittus-Boelter correlation) flow rates using water**.

To check the reliability and accuracy of fabricated experimental setup, systematic measurements were carried out using DI water as the working fluid for different flow rates. The experimental results obtained for different flow rates were correlated with well-known Shah correlation [22] and Dittus-Boelter [23] equation under the constant heat flux boundary condition. The famous Shah correlation is(6)

where *Nu *is the Nusselts number. The experimental values were reasonably in good agreement with the Shah equation as shown in Figure 8a. The same was observed for other laminar flow rates also. Reynolds number greater than 10,000 has been correlated with Dittus-Boelter equation given below:(7)

As shown in Figure 8b, the good coincidence between the experimental results and the calculated values for water reveals that the precision of the experimental system is considerably good. The uncertainty of the experimental system is less than 8%.

#### Convective heat transfer of graphene nanofluids

**Figure 9:**
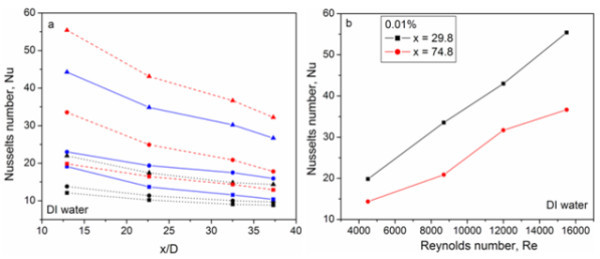
**Convective heat transfer study**. **(a) **Heat transfer measurement of f-HEG dispersed DI water based nanofluids. Black dotted lines, blue solid lines, and red dashed lines are for DI water alone, 0.005% of f-HEG and 0.01% of f-HEG, respectively. Symbols represents *Re *= 4500 (*square*), *Re *= 8700 (*circle*), and *Re *= 15,500 (*triangle*). **(b) **Measurement of Nusselts number with respect to different Reynolds numbers for DI water based nanofluids, containing 0.01% volume fraction of f-HEG.

**Figure 10:**
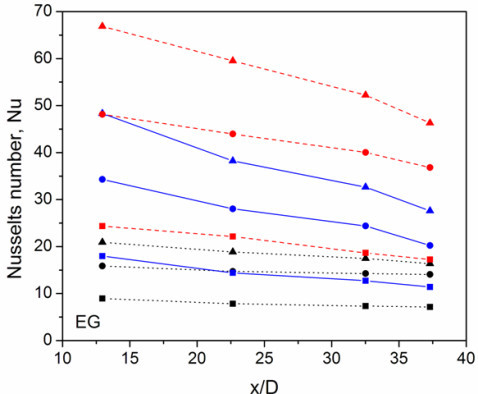
**Heat transfer measurement of f-HEG dispersed EG based nanofluids**. Black dotted lines, blue solid lines, and red dashed lines are for EG alone, 0.005% of f-HEG and 0.01% of f-HEG, respectively. Symbols represents *Re *= 250 (*square*), *Re *= 550 (*circle*), and *Re *= 1000 (*triangle*).

Having established confidence in the experimental system, systematic experiments were performed at different flow conditions (Reynolds numbers) for different f-HEG volume fractions under a constant heat flow. From the experiment heat transfer coefficient was calculated and then converts it into corresponding Nusselts number. The Reynolds number is calculated based on the viscosity of the host liquid. Since the calculated Reynolds numbers were greater than 4000, for DI water based nanofluids, the flow was considered to be turbulent. Figure 9a shows the heat transfer measurement of DI water, 0.005 and 0.01% volume fractions f-HEG dispersed DI water for different Reynolds numbers. *X*-axis shows the ratio of axial distance to diameter of the tube (*x*/*D*) and *Y*-axis the corresponding Nusselts number. Black dotted lines, blue solid lines, and red dashed lines are for DI water alone, 0.005% of f-HEG and 0.01% of f-HEG, respectively. Symbols represents *Re *= 4500 (■),*Re *= 8700 (●), and *Re *= 15500 (▲). For better understanding the change in Nusselts number for different Reynolds number is shown in Figure 9b. Similar measurements on EG based nanofluid for different volume fractions and varying Reynolds number are shown in Figure 10. Black dotted lines, blue solid lines, and red dashed lines are for EG alone, 0.005% of f-HEG and 0.01% of f-HEG, respectively. Symbols represents *Re *= 250 (■),*Re *= 550 (●), and *Re *= 1000 (▲). Since the calculated Reynolds numbers were less than 2800, for EG based nanofluids, the flow rates used were laminar.

Both the DI water and EG based nanofluids results suggests that the presence of nanomaterials dispersed nanofluids increases the Nusselts number significantly, and the increase is considerably more at high volume fractions and high Reynolds numbers. From Figure 9 it is clear that for a given f-HEG volume fraction, the Nusselts number decreases with axial distance. This is as expected for heat transfer in the entrance region. The percentage enhancement in heat transfer is calculated using the relation [*h*_n_(*x*) - *h*_f_(*x*)] × 100/*h*_f_(*x*), where *h*_f_(*x*) and *h*_n_(*x*) are the heat transfer coefficient for the base fluid and nanofluid at distance *x*, respectively. The enhancement in heat transfer for *Re *= 4500 at the tube entrance is about 64 and 76% for 0.005 and 0.01% volume fractions, respectively. At the outlet, the value decreases to about 21 and 57%, respectively, for 0.005 and 0.01%. When the Reynolds number increases (*Re *= 15,500) the enhancement also increases and it is about 108 for 0.005% and 171 for 0.01% at the entrance. At the end, the values change to about 92 for 0.005% and 141 for 0.01%, respectively.

Similar trend is observed in the case of EG based nanofluid also. Figure 10 shows the variation of Nusselts number for 0.005 and 0.01% f-HEG dispersed EG based nanofluids. From graph it is clear that heat transfer increases with volume fraction. The enhancement in heat transfer for *Re *= 250 at the tube entrance is about 100 and 172% for 0.005 and 0.01%, respectively. At the exit, the value decreases to about 59 and 140%, respectively, for 0.005 and 0.01%. Like water based nanofluids, here also the Nusselts number increases with increase in Reynolds number and it is around 130 and 219% for 0.005 and 0.01% volume fractions, respectively, at the entrance for *Re *= 1000. At the tube exit, the values change to about 69% for 0.005% and 183% for 0.01%. The enhancement in Nusselts number for EG based nanofluids are higher than that of DI water based nanofluids.

Figure 9b shows the effect of the Reynolds number on heat transfer. Figure clearly shows that the Nusselts number increases with increasing Reynolds number. There is a large difference in the Nusselts number at *Re *= 4500 and that at *Re *= 15,500 for DI water based nanofluids. Similar will be the case for EG based nanofluids also (figure not given). This suggests that Reynolds number has a significant effect on the heat transfer mechanism. The enhancement in heat transfer is very drastic compared to the enhancement in thermal conductivity. Another important observation is that even though enhancement in thermal conductivity is very low, enhancement in heat transfer is high for EG based nanofluid.

The reason for decrease in heat transfer from entrance to exit of the tube is due to the variation of thermal boundary layer. In a simple way heat transfer can be written as *k*/δ with δ the thickness of thermal boundary layer. At the entrance (*x *= 0), the theoretical boundary layer thickness is zero, hence the heat transfer coefficient approaches infinity. The boundary layer increases with axial distance until fully developed after which the boundary layer thickness and hence the convective heat transfer coefficient is constant [8]. Since there is not much enhancement in thermal conductivity, the effect of thickness of thermal boundary may be the reason for this huge enhancement in heat transfer.

Ding et al. [8] also showed that for nanofluids containing 0.5 wt% CNT, the maximum enhancement reaches over 350% at *Re *= 800 and showed that enhancement is a function of the axial distance from the inlet of the test section. Similar observations but with less significant enhancement was observed by Xuan and Li [24] in the turbulent flow regime. Wen and Ding [25] also showed similar features at the entrance region in the laminar flow regime when they investigated heat transfer of aqueous c-alumina nanofluids. They have observed around 47% increase in the convective heat transfer coefficient for 1.6 vol.% nanoparticles loading and *Re *= 1600, which is much greater than that due to the enhancement of thermal conduction (<~10%).

According to the Brownian theory [26], the smaller the sizes of the colloid particles, the faster the particles move, so that energy transport inside the liquid becomes stronger. The clustering or restacking of graphene is very less in solution. Each sheet will be separated out during ultrasonication and was well dispersed which helps for fast heat transfer. Another factor which helps in the enhancement of thermal conductivity as well as heat transfer is surface area of the material. The surface area of hydrogen exfoliated graphene is approximately 450 m^2^/g [13]. Other factors which affect the thermal conductivity and heat transfer of nanofluids are size and shape of the nanomaterials, the material (test section) in which nanofluid is flowing, temperature of the test section as well as the surrounding, viscosity of the fluid, etc. Further studies are being carried out for deeper understanding of the mechanism.

## Conclusion

Graphene was synthesized by hydrogen induced exfoliation of graphite oxide. Further we effectively dispersed f-HEG in the base fluids without any surfactant or additives by using ultrasonication. Systematic characterization and experiments were carried out for the sample preparation as well as the thermal conductivity and heat transfer measurements. The results suggest that there was considerable enhancement in thermal conductivity and heat transfer for f-HEG dispersed fluid compared to its base fluid. The Nusselts number increases with increase in volume fraction and Reynolds number of f-HEG. Similarly, the thermal conductivity of f-HEG increases due to the increase in volume fraction and temperature. Electrical conductivity of f-HEG dispersed base fluids was also showing enhancement compared to the base fluid.

## Abbreviations

DI: deionized; EG: ethylene glycol; FESEM: field emission scanning electron microscopy; GO: graphite oxide; HEG: hydrogen exfoliated grapheme; TEM: transmission electron microscopy; XRD: X-ray diffraction.

## Competing interests

The authors declare that they have no competing interests.

## Authors' contributions

TTB has carried out the experimental part, set up the experimental setup, analysis of the data and manuscript preparation. SR has participated in the design of the experimental setup,, analysis of the data and drafted the manuscript.
